# Healthcare costs associated with short-acting β_2_-agonists in asthma: observational UK SABINA study

**DOI:** 10.3399/BJGPO.2023.0015

**Published:** 2023-09-03

**Authors:** Darush Attar-Zadeh, Toby Capstick, Deborah Leese, Sofie Arnetorp, Eleni Rapsomaniki, Keith Peres Da Costa, Ekaterina Maslova, Yang Xu, Danny Gibson, Jennifer K Quint

**Affiliations:** 1 North West London Children & Young People Asthma Network, London, UK; 2 Medicines Management and Pharmacy Services, Leeds Teaching Hospital NHS Trust, Leeds, UK; 3 Sheffield Medicines Optimisation Team, NHS Sheffield Clinical Commissioning Group, Sheffield, UK; 4 Biopharmaceuticals Business Unit, Global Market Access Pricing, Vaccine & Immune Therapy, AstraZeneca, Gothenburg, Sweden; 5 Real World Science, BioPharmaceuticals Medical, AstraZeneca, Cambridge, UK; 6 AI R&D Department, ZS Associates, London, UK; 7 BioPharmaceutical Medical, Medical Affairs Respiratory and Immunology, AstraZeneca, Cambridge, UK; 8 Global Market Access and Pricing, Health Economics and Payer Evidence, AstraZeneca, Cambridge, UK; 9 National Heart Lung Institute, Imperial College London, London, UK

**Keywords:** adrenergic beta-2 receptor agonist, asthma, emergency treatment, health care costs, prescriptions, primary health care, United Kingdom

## Abstract

**Background:**

Poor asthma control is associated with high short-acting β_2_-agonist (SABA) use.

**Aim:**

To assess asthma-related healthcare resource utilisation (HCRU) and medication costs associated with high versus low SABA prescriptions in the UK.

**Design & setting:**

Analysis of SABINA I (SABA use IN Asthma I), a retrospective longitudinal study using UK electronic health records (Clinical Practice Research Datalink GOLD 2008−2019 and Hospital Episode Statistics database).

**Method:**

Eligible patients were ≥12 years old with SABA prescriptions in the past year. SABA prescriptions (number of canisters per year) were defined as high (≥3) or low (1–2). Association of SABA prescriptions with HCRU was assessed by negative binominal model adjusted for covariates. The UK unit costs from the NHS were applied to estimate total healthcare costs (2020). Medication costs were based on the annual average number of canisters per year per patient.

**Results:**

Overall, 186 061 patients with SABA prescriptions were included, of whom 51% were prescribed high SABA. Total annual average costs (HCRU and medication) were 52% higher in the high SABA group versus the low SABA group (£2 256 091 per 1000 patients/year versus £1 480 640 per 1000 patients/year). Medication costs accounted for the majority of asthma-related costs. Across both groups, most HCRU costs were for non–exacerbation-related primary care or hospital outpatient visits. The annual average HCRU cost difference for high SABA versus low SABA was the greatest for hospitalisations (+230%; £15 521 per 1000 patients/year versus £4697 per 1000 patients/year) and exacerbation-related primary care visits (+162%; £18 770 per 1000 patients/year versus £7160 per 1000 patients/year). Asthma-related HCRU extrapolated to the broader UK asthma population was £108.5 million per year higher with high SABA versus low SABA.

**Conclusion:**

High SABA versus low SABA prescriptions are associated with higher asthma-related HCRU costs.

**How this fits in**

- This study showed that high SABA inhaler use was associated with increased asthma-related HCRU in the UK, driven by non–exacerbation-related care.
- Among patients prescribed ≥1 SABA canister/year, more than half of patients were prescribed ≥3 SABA canisters/year.
- Asthma-related HCRU and medication costs were higher among patients prescribed high SABA.
- When extrapolated to the broader UK asthma population, HCRU costs were £108.5 million/year higher for patients prescribed high-SABA versus low-SABA.

**INTRODUCTION**

Asthma is a chronic disease characterised by respiratory symptoms, including shortness of breath, wheezing, chest tightness and cough,[1] and estimated to affect 339 million people worldwide,[2] with a prevalence of 6.5% in the United Kingdom (UK).[3] More than 77,000 people were hospitalised for asthma in 2016−2017, and approximately 5.4 million people are currently being treated for asthma in the UK.[4] Besides the clinical and humanistic burden on patients, asthma places a substantial economic burden on the healthcare system.[5]

Suboptimal asthma control is associated with substantial costs.[6-10] In a real-world evaluation of 462 adults from 11 European countries, the cost related to persistent asthma was €1,583 per patient (in 2010 currency), including direct and indirect costs.[11] In the UK, asthma-related costs during 2011−2012 were estimated to total ≥£1.1 billion, driven largely by primary care services (prescriptions and consultations), which accounted for 74% of the costs.[12]

Compliance with asthma management recommendations can improve asthma control.[13,14] In 2019, the Global Initiative for Asthma (GINA) stopped recommending short-acting $\beta_2$-agonist (SABA) monotherapy for as-needed symptom relief.[15] In their 2021 update,[1] GINA provides two treatment tracks: the preferred track 1 with low-dose inhaled corticosteroid (ICS)-formoterol as the preferred reliever and the alternative track 2 for when track 1 is not an option. Track 2 should only be considered if the use of ICS-formoterol as reliever is not possible and patients are likely to be adherent to their ICS-containing controller therapy, wherein as-needed SABA is recommended for symptom relief across all steps together with concomitant low-dose ICS at step 1, regular ICS at step 2 or ICS+long-acting $\beta_2$-agonist (LABA) at steps 3−5.[1] Despite current GINA recommendations, SABAs continue to be overused[16-19] by an estimated 10%−38% of the asthma population in the UK.[16,20] High SABA use is associated with decreased lung function,[21] poor asthma control,[21] increased risk of exacerbations[18,21,22] and mortality,[18] contributing to greater healthcare resource utilisation (HCRU).[22-25] The SABA use IN Asthma I (SABINA I) retrospective longitudinal observational study in >500,000 patients in the UK reported high SABA inhaler use (≥3 canisters/year) to be associated with a significant increase in asthma-related primary care and hospital outpatient consultations.[22]

This analysis of the SABINA I study evaluated the economic impact of high SABA prescriptions in the UK by estimating asthma-related HCRU and medication costs in patients across asthma treatment groups.

## METHODS

### Study design and population

The design of the overall SABINA global programme[26] and primary findings from SABINA I have been described previously.[22] Patients ≥12 years old with asthma identified between 2008 and 2019 in the Clinical Practice Research Datalink (CPRD) GOLD linked to Hospital Episode Statistics (HES) were included. Additional details are included in the supplementary section.

### Variables and outcomes

Patients were categorised by the number of SABA inhalers prescribed to them in the 12 months before the index date (defined in Supplementary Materials) as ‘low’ (1−2 SABA canisters/year; ‘low-SABA’ group) or ‘high’ (≥3 SABA canisters/year; ‘high-SABA’ group), wherein 1 canister was estimated to have an average of 150

puffs/inhaler.[22,26] Patients were classified based on ICS prescriptions according to the 2016 Scottish Intercollegiate Guidelines Network/British Thoracic Society (SIGN/BTS) guidelines into the following categories: no ICS (SABA monotherapy), low-dose ICS (400−799 μg beclomethasone dipropionate equivalent), low-dose ICS+LABA, medium-dose ICS (800−1,599 μg)±additional therapies (including LABA) and high-dose ICS (>1,600 μg)±additional therapies.[27]

Exacerbation-related HCRU was defined as asthma worsening that required a short course of oral corticosteroid (OCS; defined as a prescription for a total amount ≤300 mg) prescribed by a primary care provider or an asthma-related accident and emergency (A&E) department visit or hospital admission.[3,28,29] Non–exacerbation-related HCRU was defined as a primary care visit with no record of an OCS prescription or hospital outpatient consultations. The UK unit costs based on the National Health Service (NHS) reference costs for the healthcare services utilised for 2020 were applied to estimate HCRU costs (**Supplementary Table 1**).

Asthma medications included SABA, ICS, ICS+LABA, long-acting muscarinic antagonists, OCS, leukotriene receptor antagonists and theophylline. Drug acquisition costs were derived from the 2020 British National Formulary.[30]

**Statistical analysis**

The annual rate per patient for each asthma-related HCRU service was estimated using a negative binomial model adjusting for age, sex, comorbidities, prior exacerbations, and maintenance therapy prescriptions (proportion of days covered). Exacerbation- and non–exacerbation-related HCRU costs were quantified by multiplying the unit cost by the annual rate of each HCRU service. Medication costs

Accepted Manuscript - BJGP Open - BJGPO.2023.0015

were based on the average number of canisters per patient per year for the most prescribed drug within each drug class. The average annual number of canisters per patient was multiplied by the drug cost. HCRU and medication costs comprised the total cost of care and were stratified by SABA prescriptions and treatment group. For comparison between the two SABA prescription groups, annual costs per 1,000 patients estimated for each HCRU service or BTS treatment step were expressed as an average (i.e., sum of costs per 1,000 patients per year).

We performed crude extrapolations of HCRU costs to the overall UK asthma population and to hypothetical integrated care systems (IC)[31] and primary care network (PCN)[32,33] populations to support real-world population health decisions. We assumed a UK asthma population of 4,480,518 based on the Quality and Outcomes Framework (QOF) 2018−2020, although the QOF includes patients <12 years old[34] and our analysis did not. The hypothetical 'average' IC and PCN populations were estimated to comprise 1,700,000 and 50,000 patients, respectively. Population distribution assumptions from the CPRD cohort were applied to these populations for the extrapolation exercise.

To understand the influence of adherence, a sensitivity analysis was conducted to evaluate asthma-related HCRU costs among patients with ≥50% proportion of days covered (PDC) with prescribed ICS. Evaluation of the sample size showed that the majority of patients had PDC <50%; the ≥50% threshold was used to ensure a sufficient sample for analysis.

Accepted Manuscript - BJGP Open - BJGPO.2023.0015

**RESULTS**

A total of 235,946 patients ≥12 years old with asthma were identified in the UK CPRD GOLD-HES-linked cohort (2008−2019). Of these, 49,885 (21%) patients without SABA prescriptions were excluded, leaving 186,061 (79%) with SABA prescriptions eligible for the primary economic analysis (**Figure 1**). Over half of included patients (57%) received no ICS or low-dose ICS prescriptions. Overall, 94,544 (51%) patients were categorised in the high-SABA group (≥3 canisters/year); the proportion of patients with high-SABA prescriptions increased with increasing asthma treatment step, from 28% in the group with no ICS to 77% in the group with high-dose ICS±additional therapies.

**Asthma-related HCRU rates and costs**

Across all treatment steps, rates of HCRU were higher in the high- versus low-SABA group. Rates were particularly higher among those in the high-SABA group who were receiving medium- or high-dose ICS with or without additional therapies (**Supplementary Tables 2 and 3**). Patients in the high-SABA group (£474,794/1,000 patients/year) had 69% greater annual average HCRU costs than the low-SABA group (£280,280/1,000 patients/year) (**Figures 2A, 2B, 3A, 3B**). The difference in annual average HCRU costs between low- and high-SABA groups was most pronounced (additional 77%) among those receiving medium-dose ICS±additional therapies (**Figure 3B**). Total annual HCRU-related costs were £7,910,112 and £3,402,384 for high- and low-SABA groups, respectively, with non–exacerbation-related HCRU accounting for most of the costs in both groups (54.7% and 64.1%, respectively; **Figure 4**). Non–exacerbation-related HCRU costs were higher for the high- versus low-SABA group (**Figure 2A**). Among non–exacerbation-

Accepted Manuscript - BJGP Open - BJGPO.2023.0015

related encounters, primary care visits without OCS in patients not receiving ICS or receiving low-dose ICS, and outpatient visits in patients receiving medium- or high-dose ICS±additional therapies accounted for most HCRU costs in both SABA groups (**Supplementary Figure 1**). Across treatment steps, exacerbation-related HCRU accounted for 3.8%−19.6% of high-SABA HCRU costs and 3.1%−16.9% of low-SABA HCRU costs (**Figure 4**). Exacerbation-related HCRU costs were higher in the high- versus low-SABA group, with the greatest difference observed for hospitalisations (difference in cost: £10,824; 230%), followed by primary care consultations with OCS prescriptions (£11,610; 162%) and A&E visits (£2,092; 141%; **Figure 2B**).

**Asthma-related medication costs**

Overall, medication costs accounted for the majority of asthma-related costs (**Figure 5A**) and increased with increasing treatment steps. Total annual average medication costs were 48% higher in the high- versus low-SABA group (£1,781,297 versus £1,200,359/1,000 patients per year; **Supplementary Figure 2**). Maintenance medications accounted for nearly all medication costs across all levels of asthma treatment (low SABA, 87%−100%; high SABA, 78%−98%; **Supplementary Figure 3**).

**Total asthma-related costs**

Total annual costs were £38,705,967 and £13,906,864 for the high- and low-SABA groups, respectively. Total of the annual average costs were 52% greater for high-SABA (£2,256,091/1,000 patients per year) versus low-SABA (£1,480,640/1,000 patients per year) across all treatment groups (**Figure 5A**). The cost increased with

Accepted Manuscript - BJGP Open - BJGPO.2023.0015

increasing treatment steps and the percentage increase in total costs between the high-SABA and low-SABA groups was particularly high among those with no ICS (88%) or low-dose ICS (93%; **Figure 5B**).

**Extrapolation of asthma-related HCRU to UK, IC and PCN populations**

HCRU costs derived from the primary analyses and extrapolated to a broader UK asthma population (based on the QOF database) were estimated to be £108,550,179/year higher for the high-SABA versus low-SABA group (total of £190,482,678 versus £81,932,499/year; **Supplementary Table 4**). The proportionately higher HCRU in the high-SABA group translated to an additional £41,186,153/year and £1,211,357/year in the IC and PCN populations, respectively, versus the extrapolated low-SABA group (**Supplementary Tables 5 and 6**).

**Exploratory sensitivity analysis of HCRU costs in patients with ≥50% PDC**

A total of 67,379 (36%) patients with SABA prescriptions had ≥50% PDC for ICS prescriptions and were included in the exploratory sensitivity analysis; 26,526 (14%) had PDC 50−75%, and 40,853 (22%) had PDC ≥75%. Most patients with ≥50% PDC (75%) were in the high-SABA group; the majority (65%) received low-dose ICS (42%) or low-dose ICS+LABA (23%). The relative differences in HCRU costs between high- and low-SABA groups were comparable to the full analysis cohort among patients prescribed low-dose ICS, but smaller among patients prescribed medium- and high-dose ICS (**Supplementary Figure 4**).

## DISCUSSION

### Summary

Accepted Manuscript - BJGP Open - BJGPO.2023.0015

In this study involving patients with asthma in a UK cohort who received SABA prescriptions, half of all patients were prescribed a high volume of SABAs (≥3 canisters/year), which was associated with higher asthma-related HCRU, medication, and total costs. Overall, medication costs accounted for most of the asthma-related costs. Regardless of the type of HCRU, patients in the high-SABA versus the low-SABA group incurred greater HCRU costs, particularly hospitalisations. However, overall differences in HCRU costs were driven by non–exacerbation-related HCRU. Total of the annual average costs were 52% higher for patients with high-SABA versus low-SABA and particularly high for those who did not receive ICS or received low-dose ICS; suggestive of poorly controlled disease even in patients with mild asthma. When our findings were extrapolated to an estimated 4.5 million patients with asthma and SABA prescriptions in the broader UK population, higher SABA prescriptions translated to an estimated additional £108.5 million per year in asthma-related HCRU. This study highlights the difference in healthcare costs between patients with high- and low-SABA prescriptions and therefore, the potential savings that may be achieved by optimising care through implementation of the latest evidence-based guidelines targeting reductions in SABA use.

**Strengths and limitations**

A major strength of this study is the use of the CPRD GOLD database, one of the largest primary care databases in the world,[35] enabling generalisation of our findings to a wider UK population.

This study has some limitations. The exclusion of patients who were not prescribed SABA may have resulted in the omission of well-controlled patients with asthma with low HCRU; however, this is expected to have a minimal impact on our findings.

Accepted Manuscript - BJGP Open - BJGPO.2023.0015

Patients receiving ICS-formoterol reliever only and untreated patients may also have been excluded owing to the above criterion. Notably, records of SABA prescriptions may not always reflect actual use, which cannot be captured in the data sources. Furthermore, asthma management guidelines recommend that follow-ups be considered in patients with exacerbations;[27,31] however, these were not included in the exacerbation-related costs. Although high SABA use can be a marker of poor disease control,[36] our analysis precluded determination of the cause-effect relation between SABA prescriptions and asthma exacerbations. Therefore, this study shows an association, and not causality, between high SABA prescriptions and high HCRU. The association of asthma related HCRU and costs with higher SABA prescriptions (beyond the cut-off for ≥3 canisters) was not analysed. However, owing to lack of consensus on appropriate use of rescue therapy[37] and what level of SABA use constitutes "excessive" exposure, an evidence-based binary classification of SABA prescriptions (≥ 3 vs 1–2 canisters/year) may offer a practical approach to addressing patients' reliance on SABAs in clinical practice.

The threshold of ≥50% PDC was selected to ensure a sufficient sample size for the sensitivity analysis of HCRU costs (n=67,379; 36%) and enable a meaningful comparison between the SABA prescription groups; overall adherence to ICS among patients with asthma is reported to be ~50%.[38] In other settings, higher PDC (≥75%−80%) may suggest better adherence but we were unable to conduct the sensitivity analysis with higher PDC thresholds because of the available sample size (n=40,853; 22%). The observational analysis of 10 SABINA datasets involving 1,033,564 patients with asthma from North American and European countries, including the UK, also utilised the threshold of ≥50% PDC, revealing that only 39.4% of GINA step 2−5-treated population received maintenance therapy 50% or more of

Accepted Manuscript - BJGP Open - BJGPO.2023.0015

the time,[39] which is comparable to our findings. Finally, the extrapolation exercise was meant to be a conceptual illustration rather than a prediction of actual costs for a specific entity and should be interpreted as such.

**Comparison with existing literature**

Previous studies have illustrated the high burden and costs of asthma management in the UK.[12,22,40] While these studies did not address costs associated with SABA prescriptions, our study highlights higher asthma-related healthcare costs (HCRU, medication costs and total costs) with high-SABA versus low-SABA prescriptions. Results from two large database studies in the United States (US) and the UK showed that total HCRU was highest in patients with severe (GINA step 5) versus mild (GINA step 1) asthma.[41] Our results are consistent with these studies and we observed particularly high HCRU costs for high SABA among patients receiving medium-dose ICS±additional therapies.

An administrative claims analysis in the US (2013−2014) showed that patients with asthma at GINA steps 4−5 incurred significantly greater direct and indirect costs driven by exacerbations and rescue medication use versus those at GINA steps 1−3.[42] We observed that while maintenance medications accounted for nearly all medication costs across all treatment levels, the costs for both rescue and maintenance medications were higher for the high-SABA versus low-SABA group.

A retrospective study of more than 90,000 patients with asthma in the US (2003−2007) found that high (6.5−12 canister equivalents (CE)) or excessive (>12 CEs) SABA use was associated with significantly higher emergency department (ED) visits, hospitalisations and OCS use and subsequently higher healthcare costs than low-SABA use (0.5−2 CEs).[23] Mean total asthma-related costs per patient/year were 2.2 times ($1,326 [95% confidence interval (CI): $1,256, $1,395]) and 3 times

Accepted Manuscript - BJGPO.2023.0015

($1,791 [95% CI: $1,670, $1,913]) higher in high and excessive users, respectively, versus low users ($889 [95% CI: $866, $913]).[23] Similarly, mean per patient all-cause healthcare costs were higher in the high ($5,146 [95% CI: $4,874, $5,419]) and excessive ($5,962 [95% CI: $5,461, $6,463]) users versus the low ($4,777 [95% CI: $4,609, $4,944]) users.[23] While we did not evaluate the same thresholds of high SABA prescriptions (we categorised high SABA prescriptions as ≥3 canisters/year), our findings were generally consistent with these previous reports.

Only one-third of all patients with SABA prescriptions had ≥50% PDC for ICS prescriptions, suggesting suboptimal annual coverage for ICS-containing medications. Our findings are consistent with the results of a systematic review, reporting that very low adherence to ICS-containing medications was common and consistently associated with a higher risk of severe asthma exacerbations.[43] Likewise, the European Community Respiratory Health Survey which studies asthma treatments over a 20-year period (1991–1994, 1998–2002, and 2011–2014) in 11 countries, including the UK, revealed that only 34% of patients with persistent disease take ICS on a regular basis.[44] Additionally, analysis of general practitioner prescription refill records of 182 patients with difficult asthma (GINA steps 4−5) treated at a specialist clinic in Northern Ireland revealed that 35% of patients filled 50% or fewer prescriptions for ICS-containing combination medications, with 88% admitting poor adherence with inhaled therapy.[45]

**Implications for research and/or practice**

High SABA prescriptions and usage are common[16-19,46] despite established associations with increased risk of exacerbations, asthma-related hospitalisations and ED visits.[24,25,46] Furthermore, patients with mild asthma using high SABA

Accepted Manuscript - BJGP Open - BJGPO.2023.0015

experience a 20% increase in exacerbations versus those using low SABA.[22] Our study has focused on the translation of known clinical implications to HCRU and costs. Increased asthma-related HCRU and medications costs associated with high SABA prescriptions contribute to the overall economic burden of asthma to the healthcare system in the UK. Considering GINA no longer recommends treatment with as-needed SABA without concomitant ICS for patients ≥12 years of age,[1] associated medical costs may be averted by aligning clinical practices with guidelines to achieve asthma control.[47] This approach may subsequently provide meaningful reductions in healthcare expenditures at the local and national levels. Additionally, conducting localised audits for high-SABA users and prioritising provision of a Medicines Optimisation team to address poor asthma management are recommended.

Worryingly, our findings also show that adherence to ICS-containing medications remains suboptimal in many patients with asthma. Indeed, real-world adherence to asthma medications, particularly ICS, remains low, increasing the risk of poor patient outcomes.[38,43] Conversely, adherence with ICS treatment in adults with asthma has been shown to reduce the risk of exacerbations.[48] Of note, findings from a systematic literature review of 19 studies, involving 26,563 patients with asthma, revealed that 24% of exacerbations and 60% of related hospitalisations could be attributed to poor adherence to ICS-containing medications.[48] Considering the UK National Review of Asthma Deaths report identified high SABA use and insufficient provision of ICS-containing medications as preventable causes of asthma deaths,[49] there is a need to regularly monitor SABA prescriptions and suboptimal adherence to controller therapy. It is also essential that physicians build strong partnerships with patients through shared decision-making[36] to improve both adherence[50] and

Accepted Manuscript - BJGP Open - BJGPO.2023.0015

treatment outcomes,[50,51] and provide training on effective self-management to reduce HCRU.[52]

Accepted Manuscript - BJGP Open - BJGPO.2023.0015

**Funding:** This study was funded by AstraZeneca.

**Ethical approval**

The Independent Scientific Advisory Committee (ISAC) for the Medicines and Healthcare products Regulatory Agency database research approved the research protocol (protocol number 18_080R). A Health Research Authority Research Ethics Committee (East Midlands–Derby, REC reference number 05/MRE04/87) provided generic ethical approval for observational research using CPRD with approval from ISAC. CPRD provided pseudonymised data for this study, which were linked by NHS Digital. This process was approved by select practices; individual patients had the right to opt out.

**Competing interests**

DA-Z has served on advisory boards and reports speaker fees from AstraZeneca, Boehringer Ingelheim, Chiesi, Cipla, GlaxoSmithKline, Glenmark, Janssen, Johnson & Johnson, Napp, Novartis, Orion, Pfizer, Scope, Teva, Thornton & Ross, Trudell and Viatris. TC reports nonfinancial support from Napp Pharmaceuticals and other support from AstraZeneca, Boehringer Ingelheim, Chiesi, GlaxoSmithKline, Insmed and Novartis outside the submitted work. DL reports speaker fees and conference attendance fees from AstraZeneca and Chiesi. SA, HvH, ER, KPDC, EM and YX are employees of AstraZeneca. JKQ's research group received funds from AstraZeneca for some of this work, and JKQ received speaker fees from AstraZeneca.

**Acknowledgements**

Medical writing and editorial support was provided by Suchita Nath-Sain, PhD, and Jeff Frimpter, MPH, of Cactus Life Sciences (part of Cactus Communications) in accordance with Good Publication Practice (GPP3) guidelines (http://www.ismpp.org/gpp3). This support was fully funded by AstraZeneca.

Accepted Manuscript - BJGP Open - BJGPO.2022.0015

**FIGURES**

**Figure 1:** Patient disposition and distribution of UK patients with asthma based on SABA inhaler use and BTS treatment steps

*Hospital admissions and outpatient consultation data were obtained from the HES database. HES only covers hospitals in England.

BTS, British Thoracic Society; CPRD, Clinical Practice Research Datalink; ICS, inhaled corticosteroid; HES, Hospital Episode Statistics; LABA, long-acting $\beta_2$-agonist; SABA, short-acting $\beta_2$-agonist.

**Figure 2:** HCRU service costs in UK patients with asthma for SABA ≥3 versus SABA 1−2 (A) Per 1,000 patients* (B) Cost differential: high-SABA versus low-SABA group (absolute and percentage differences)

*Expressed as an average (i.e., sum of costs per 1,000 patients per year).

A&E, accident and emergency; BTS, British Thoracic Society; HCRU, healthcare resource utilisation; OCS, oral corticosteroid; SABA, short-acting $\beta_2$-agonist.

**Figure 3:** HCRU costs in UK patients with asthma for SABA ≥3 versus SABA 1−2 based on BTS treatment steps (A) Per 1,000 patients* (B) Cost differential: high-SABA versus low-SABA group (absolute and percentage differences)

Accepted Manuscript - BJGP Open - BJGPO.2023.0015

*Expressed as an average (i.e., sum of costs per 1,000 patients per year). Estimates are subject to rounding

BTS, British Thoracic Society; HCRU, healthcare resource utilisation; ICS, inhaled corticosteroid; LABA, long-acting $\beta_2$-agonist; SABA, short-acting $\beta_2$-agonist; UK, United Kingdom.

**Figure 4:** Costs for exacerbation- and non–exacerbation-related HCRU in UK patients with asthma by BTS treatment steps

Data presented as an average (i.e., sum of costs per 1,000 patients per year). Primary care visits included physician and nurse clinic, outpatient, telephonic and home visits.

A&E, accident and emergency; BTS, British Thoracic Society; HCRU, healthcare resource utilisation; ICS, inhaled corticosteroid;

LABA, long-acting $\beta_2$-agonist; OCS, oral corticosteroid; SABA, short-acting $\beta_2$-agonist.

**Figure 5:** Asthma-related HCRU and medication costs in UK patients with asthma by BTS treatment steps (A) Per 1,000 patients* (B) Total cost differential: high-SABA versus low-SABA group (absolute and percentage differences)

*Expressed as an average (i.e., sum of costs per 1,000 patients per year). Estimates are subject to rounding. In panel A, lighter shades represent data for the low-SABA group, while darker shades represent data for the high-SABA group.

Accepted Manuscript - BJGP Open - BJGPO.2023.0015

BTS, British Thoracic Society; HCRU, healthcare resource utilisation; ICS, inhaled corticosteroid; LABA, long-acting $\beta_2$-agonist; SABA, short-acting $\beta_2$-agonist; UK, United Kingdom.

Accepted Manuscript - BJGP Open - BJGPO.2023.0015
